# Regulation of PKC Mediated Signaling by Calcium during Visceral Leishmaniasis

**DOI:** 10.1371/journal.pone.0110843

**Published:** 2014-10-17

**Authors:** Nivedita Roy, Supriya Chakraborty, Bidisha Paul Chowdhury, Sayantan Banerjee, Kuntal Halder, Saikat Majumder, Subrata Majumdar, Parimal C. Sen

**Affiliations:** Division of Molecular Medicine, Bose Institute, Kolkata, West Bengal, India; University of KwaZulu-Natal, South Africa

## Abstract

Calcium is an ubiquitous cellular signaling molecule that controls a variety of cellular processes and is strictly maintained in the cellular compartments by the coordination of various Ca^2+^ pumps and channels. Two such fundamental calcium pumps are plasma membrane calcium ATPase (PMCA) and Sarco/endoplasmic reticulum calcium ATPase (SERCA) which play a pivotal role in maintaining intracellular calcium homeostasis. This intracellular Ca^2+^ homeostasis is often disturbed by the protozoan parasite *Leishmania donovani*, the causative organism of visceral leishmaniasis. In the present study we have dileneated the involvement of PMCA4 and SERCA3 during leishmaniasis. We have observed that during leishmaniasis, intracellular Ca^2+^ concentration was up-regulated and was further controlled by both PMCA4 and SERCA3. Inhibition of these two Ca^2+^-ATPases resulted in decreased parasite burden within the host macrophages due to enhanced intracellular Ca^2+^. Contrastingly, on the other hand, activation of PMCA4 was found to enhance the parasite burden. Our findings also highlighted the importance of Ca^2+^ in the modulation of cytokine balance during leishmaniasis. These results thus cumulatively suggests that these two Ca^2+^-ATPases play prominent roles during visceral leishmaniasis.

## Introduction

*Leishmania donovani,* an obligatory intracellular protozoan parasite which resides and multiplies within the host macrophages [1], is the causative agent of visceral leishmaniasis or Kala-azar, a fatal disease which is endemic in many parts of the tropical world [2], [3]. For effective parasite clearance, activated macrophages induce various host-protective immune responses [4]. To counteract these anti-*Leishmania* responses, the parasites induce various immune-silencing mechanisms for its intracellular survival inside the hostile environment of macrophages. Impairment of the classical protein kinase C (PKC) signaling within the host macrophages is one of the major manipulative strategies through which the parasite exerts its immunosuppressive effects.

PKC is structurally related to serine-threonine family. On the basis of structural and regulatory properties, PKC has been grouped into three subfamilies [5], [6]; conventional or classical (c-PKCs), novel (n-PKCs), and atypical (a-PKCs). Out of these three families, only conventional PKCs (α, βI, βII, and γ) require Ca^2+^ along with DAG and phosphatidylserine for their activation.

Calcium plays crucial roles in different physiological, biochemical and signaling pathways in both animal and plant cells. Moreover, calcium is one of the most widespread second messengers involved in several signal transductions. Intracellular organelles like mitochondria and endoplasmic reticulum, are the store house of calcium; they constantly accumulate and release calcium during certain cellular events to maintain the calcium homeostasis [6]. Calcium ions enter the cytoplasm either from outside the cell through the cell membrane calcium channels or from some internal calcium storages such as endoplasmic reticulum and mitochondria. On the other hand, Sarco-endoplasmic reticulum Ca^2+^-ATPase resides in the sarcoplasmic reticulum (SR) which is involved in the transport of Ca^2+^ from the cytosol to the lumen of the SR at the expense of ATP hydrolysis during muscle relaxation. The Plasma membrane Ca^2+^-ATPase (PMCA4) also obtains energy to pump calcium out of the cell by hydrolysing adenosine triphosphate (ATP).

Several studies have implicated the role of PKCs in the control of intracellular microbial replication. In this context, the *Leishmania* parasite has achieved a great deal of attention because it impairs Ca^2+^-dependent PKC signaling in the infected macrophages [7]. Infection of macrophages with lipophosphoglycan (LPG), a *Leishmania donovani* derived glycolipid, leads to the down-regulation of classical PKC activity along with upregulation of Ca^2+^-independent atypical PKC-ζ expression [7]. The selective upregulation of calcium independent PKC activity enables the parasites to survive within the hostile macrophage-microenvironment [8]. Moreover, recent findings suggest that calcium dependent calmodulin kinase activation helps in the establishment of the pathogen, *Leishmania donovani*, within the host macrophages [2], [9], [10], [11].

In the present communication, we were prompted to study the role of PMCA4 and SERCA3 in visceral leishmaniasis and found that both Ca^2+^-ATPases were induced in *Leishmania* infected macrophages. Moreover, the enhanced PMCA4 expression was found to increase the parasite burden. Similar result was obtained upon thapsigargin treatment, a SERCA3 inhibitor. In contrast, enhancement of intracellular calcium by ionomycin treatment induced the expression of calcium dependent PKCs. This in turn was found to regulate the profile of TH1 and TH2 cytokines differentially. Altogether, these results clearly suggested that the enhanced intracellular calcium was regulated by both PMCA4 and SERCA3 during visceral leishmaniasis.

## Materials and Methods

### Reagents and Chemicals

Dulbecco’s Modified Eagle’s Medium (DMEM), M-199 medium (M199), penicillin, streptomycin, EGTA and ionomycin were purchased from Sigma Chemicals (St Louis, MO, USA). Fetal calf serum (FCS) was obtained from Gibco BRL Grand Island, NY, USA and ELISA Assay Kit (Quantikine M) for tumor necrosis factor-α (TNF-α), Interferon-γ (IFN-γ), IL-12, IL-10 were from R&D Systems (Minneapolis, MN, USA).

### Parasites maintenance

*L. donovani* organisms (strain MHOM/IN/1983/AG-83) were maintained in Medium 199 (Sigma) plus 10% fetal calf serum (Gibco).

### Maintenance of RAW 264.7

RAW 264.7 macrophages were maintained in complete Dulbecco's modified Eagle's medium (SIGMA) containing 10% FCS.

### *In vitro* parasite burden determination

Macrophages were plated on eight well chamber slides (10,000 cells/well) and were treated for 30 min with inhibitors of Sarco-endoplasmic Reticulum Calcium ATPase (SERCA3) (1 µM thapsigargin) and Plasma Membrane Ca^2+^-ATPase (PMCA4) (10 µM trifluoperazine). These were then infected with *L. donovani* in a 1∶10 ratio. Unbound parasites were washed off after 4 hr. Cells were then incubated for 24 hr, fixed with chilled methanol, and stained with Giemsa following of which amastigotes were microscopically counted.

### Isolation of plasma membrane fraction

Cells were scraped using chilled PBS and centrifuged at 2000 rpm for 10 min at 4°C. The pellet was resuspended in 100 µl of extraction buffer containing 50 mM Tris-HCl, 50 mM EDTA, 1 mM PMSF, 50 mM β-ME and protease inhibitors and kept on ice for 30 min. Cells were sonicated and spun at 100000 g for 35 min at 4°C. The pellet was collected and resuspended in 100 µl of extraction buffer containing 50 mM Tris-HCl, 50 mM EDTA, 1 mM PMSF, 50 mM β-ME, protease inhibitors including 0.2% NP-40. The cell suspension was vortexed at 4°C for 30 min and centrifuged at 12,000 rpm for 10 min. Supernatant was collected and used for the measurement of PMCA4 activity.

### Plasma membrane calcium ATPase activity assay

The method described in this protocol is a modified one from the procedure reported by Sarkadi *et.al* [12]. 5 µg of membrane protein was incubated in a medium containing 30 mM Histidine, 1 mM CaCl_2_, 1 mM MgCl_2_, and 1 mM ATP for 30 min. The reaction was arrested at 45 min by the addition of 8% (final concentration) cold tri-chloro-acetic-acid (TCA). Tubes were vortexed and 40 mM of ammonium molybdate together with 2% ascorbic acid was added and incubated at room temperature for 10 min and the absorbance was measured at 820 nm.

### Measurement of cytosolic calcium

Control and infected macrophages were scrapped from the plate using chilled PBS. Cells were centrifuged and pellet dissolved in calcium free buffer (2 ml 1M HEPES, 0.5 ml 20% glucose, 20 ml HBSS). Cells were counted by hemocytometer. 3 µl of Fura-2/AM (3 µl of 1.5 mM Fura-2/AM, 2 µl of 20% Puronic F-127, 10 µl of 10% BSA) was added in 1 ml cell solution at 37°C for 30 min. Cells were centrifuged and the pellets were dissolved in calcium free buffer and transferred in quartz cuvette. After 60 sec., one set of cells were stimulated with 1 µM thapsigargin followed by 10 µM trifluoperazine. In another set of cells, after 90 sec., ionomycin (1 µM) stimulation was given followed by 50 µM EGTA. At indicated time points, samples were analyzed by flow cytometry (F = 340/380).

### Cytokine enzyme-linked immunosorbent assay (ELISA)

The conditioned medium of macrophage culture was assayed for mouse cytokines by the sandwich ELISA kit (BD Biosciences). The assay was performed according to the manufacturer’s instructions.

### Preparation of cell lysate and immunoblot analysis

The adherent cell population was scraped and centrifuged at 3000 RPM for 15 min at 4°C. The cells were then resuspended in ice-cold extraction buffer containing 10 mM Tris-HCl (pH 7.5), 4.5 mM EGTA, 2.5 mM EDTA, protease inhibitor mixture, and 1 mM Na_3_VO_4_. The protease inhibitor mixture consisted of 0.33 mM leupeptin, 0.2 mM phenyl-methyl-sulfonyl-fluoride (PMSF), 0.35 mM antipain, 0.24 mg of chymostatin per ml, 0.35 mM pepstatin, and 4.8 TIU of aprotinin per ml. The suspension was sonicated at 4°C and centrifuged at 7000 RPM for 10 min at 4°C, and the supernatant was used for the experiments. An amount of 40 µg protein was subjected to 10% sodium dodecyl sulfate polyacrylamide gel electrophoresis, and was subsequently transferred to a nitrocellulose membrane. The membrane was blocked overnight with 3% bovine serum albumin in Tris-saline buffer (pH, 7.5), and immunoblotting was performed to examine the expression levels of SERCA3, PMCA4, PKC-β and -ζ.

### Isolation of RNA and Reverse Transcription PCR

RNA was isolated according to the standard protocol [13]. Briefly, total RNA extracted from macrophage (TRI reagent; Sigma) was reverse transcribed using Revert Aid M-MuLV reverse transcriptase (Fermentas). The cDNA encoding the SERCA3, PMCA4, PKC-β,-ζ, IL-10, IL-12, TNFα, IFN-γ and GAPDH gene was amplified using specific primers as listed in Table 1. PCR amplification was conducted in a reaction volume of 50 µl using a Perkin Elmer Gen Amp PCR system 2400 and 0.5 unit of Taq polymerase set for 35 cycles (denaturation: at 94°C for 30 s; annealing: temperature vary for different primers for 30 s; extension: at 72°C for 30 s). PCR amplified product was subsequently size fractioned on 1.5% agarose gel, stained with ethidium bromide and visualized under UV-light.

**Table 1:** Sequences of the PCR primers.

GAPDH	FW- 5-GTT GTC TCC TGC GAC TTC AAC A-3 RV- 5-TCT CTT GCT CAG TGT CCT TGC T-3
PKCβ	FW- 5-CAT ATC AGC CAG TCT CCG TTA G-3 RV- 5-CTC TCC CTT CCT TCC TTT CTT C-3
PKCζ	FW-5-CGA TGG GGT GGA TGG GAT CAA AA-3 RV-5-GTG TTC ATG TCA GGG TTG TCC G-3
PMCA4	FW-5-GGA GAA AGG GAA GGA AGC ATA A-3 RV-5-CCA GGT GGC AGT GTG TAA ATA-3
SERCA3	FW-5-TGG ACC CTG CTG AAC ATA AC-3 RV-5-GAG CCT CAA AGG TGG GAA TAG-3
IL10	FW-5-ACT TGG GTT GCC AAG CCT TAT-3 RV-5-ATC ACT CTT CAC CTG CTC CAC T-3
IL12	FW-5-ATT GAA CTG GCG TTG GAA GCA-3 RV-5-TGC GCT GGA TTC GAA CAA AGA-3
TNFα	FW-5-ACG TCG TAG CAA ACC ACC AA-3 RV-5-TGA GAT AGC AAA TCG GCT GAC G-3
IFNγ	FW-5-AGC TCT TCC TCA TGG CTG TTT C-3 RV-5-TGT TGC TGA TGG CCT GAT TGT-3

### Densitometry analysis

Immunoblots and PCR products were analyzed using a model GS-700 Imaging Densitometer and Molecular Analyst (version 1.5; Bio-Rad Laboratories).

### Statistical analysis

In vitro cultures were done in triplicate. Data, shown as means ± standard deviations, from one experiment performed at least three times. The Student *t* test was used to assess the significance of differences between the mean values for the control and experimental groups. A difference with P<0.05 was considered significant, a difference with *P*<0.001 was considered highly significant and ns implied non-significant.

## Results

### Measurement of cytosolic calcium during visceral leishmaniasis

**Figure 1:**
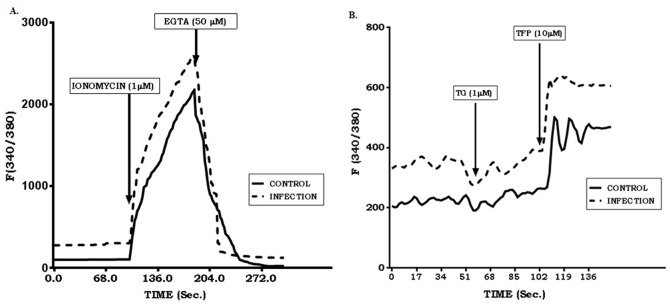
Detection of intracellular Ca^2+^ in Leishmania donovani infected macrophages in presence of different chemical agents. Uninfected (2×10^6^ cells) and *Leishmania donovani* infected macrophages (1∶10) were treated with Fura-2/AM (5 µM). Geometric mean fluorescence intensity was monitored at different time intervals by flow cytometry with the addition of ionomycin (1 µM), EGTA (50 µM) (A) and thapsigargin (TG, 1 µM), trifluoperazine (TFP, 10 µM) (B). Data shown is a representative one from three different experiments conducted under identical conditions.

*Leishmania donovani* infection is associated with abrogated calcium dependent PKC activation in macrophages [8]. Therefore, we investigated the intracellular calcium level in macrophages during *Leishmania donovani* infection. The addition of a calcium ionophore (1 µM), ionomycin, [14] provoked a rapid and sustained increase in the intracellular calcium concentration inside the control as well as infected macrophages. Upon removal of extracellular Ca^2+^ using EGTA (50 µM), the fast initial increase in response to ionomycin disappeared but slower sustained elevation in cytosolic Ca^2+^ was observed (Fig. 1A). The addition of thapsigargin (1 µM), a SERCA3 inhibitor [15], slowed down (5–10 fold) the intracellular calcium concentration in the control and infected macrophages, while the addition of trifluoperazine (10 µM), a PMCA4 inhibitor [16], intracellular calcium level was increased and sustained for a while (Fig. 1B). Interestingly, in all cases of *Leishmania donovani* infected macrophages, a significant level of increase in the intracellular calcium ion concentration (20–80 folds higher as compared to control untreated macrophages) was observed. Therefore, our results indicated that the intracellular calcium ion release is relatively high in *Leishmania donovani* infected macrophages.

Moreover, SERCA3 blocking resulted in a transient decrease in intracellular Ca^2+^ level, whereas, blocking of PMCA4 with TFP resulted in a sharp and sustained elevation in intracellular Ca^2+^ concentration.

### PMCA4 and SERCA3 expression in macrophages during Leishmania donovani infection

**Figure 2:**
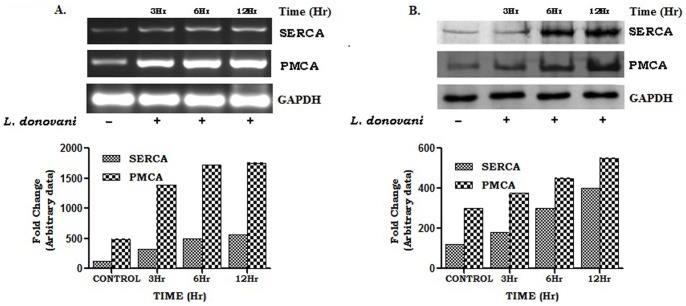
Expression of SERCA3 and PMCA4 at different time periods of infection. Macrophages (2×10^6^ cells) were infected with *L. donovani* (1∶10) and incubated at 37°C for 3, 6 and 12 hr. The cells were collected in Trizol for RNA extraction and semi-quantitative RT-PCR was performed. PMCA4, SERCA3 and GAPDH PCR products were resolved on an agarose gel (1.5%) and quantified densitometrically using lab software as described in Methods (A). In a separate set of experiment, cell lysates were prepared from infected macrophages, followed by Western blot for PMCA4, SERCA3 and GAPDH (B). Data shown above (A and B) is a representative one experiment, performed at least three times.

The intracellular calcium ion concentration is primarily regulated by two Ca^2+^-ATPases, PMCA4 and SERCA3. Therefore, we analyzed the protein and mRNA expression status of these two ATPases in *Leishmania donovani* infected macrophages. PMCA4 and SERCA3 both have several isoforms. PMCA4 activation relates to PKC isotypes, where as SERCA3 is ubiquitous. We therefore have selected both of them in our study. The expression of PMCA4 and SERCA3 was examined in *Leishmania donovani* infected macrophages at different time points of infection. The results showed that both the SERCA3 and PMCA4 expression gradually increased from 3 to 12 hr of post infection (Fig. 2A, B).

### Effect of ionomycin and EGTA on the modulation of PKCs and expression of Ca^+^- ATPases in macrophages during Leishmania donovani infection

**Figure 3:**
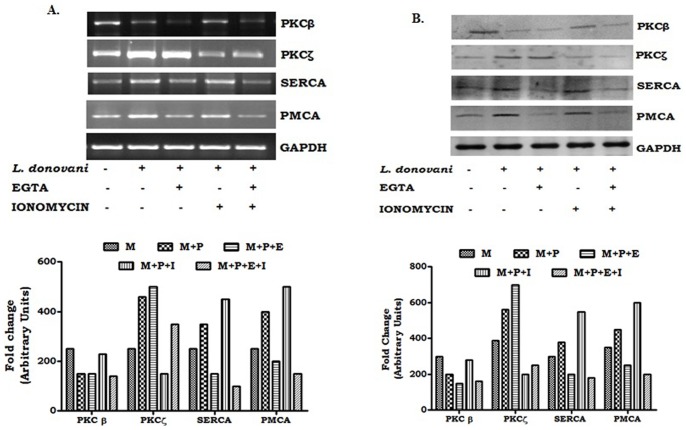
Effects of EGTA and ionomycin on mRNA and protein expression of PKC-β, PKC-ζ, SERCA3 and PMCA4 in Leishmania-infected macrophages. Macrophages (2×10^6^ cells) were infected with *L. donovani* (1∶10) and with 2 mM EGTA and/or 1 µM Ionomycin respectively. After 4 hr, cells were washed and incubated for another 3 hr at 37°C. The cells were collected in Trizol for RNA extraction and semi-quantitative RT-PCR was performed. PKC-β, PKC-ζ, SERCA3, PMCA4, and GAPDH PCR products were resolved on an agarose gel (1.5%) and quantified densitometrically using lab software as described in Methods (A). In a similar experiment, cell lysates were prepared from EGTA and/or ionomycin treated infected macrophages, followed by Western blot for PKC-β, PKC-ζ, SERCA3, PMCA4, and GAPDH (B). Data presented above (A and B) is a representative one of three experiments conducted under identical conditions.

It is well known that conventional PKC activity is strictly dependent upon the intracellular calcium ion concentration [17], whereas, atypical PKC does not require Ca^2+^ for activation [18]. Therefore, the involvement of calcium in the regulation of protein and mRNA expression of different PKC isotypes was investigated in *Leishmania donovani* infected macrophages. We observed a significant down-regulation of PKC-β (conventional PKC) protein expression in infected macrophages compared to that of the uninfected control macrophages. To study the role of Ca^2+^, an elevator, ionomycin and a chelator, EGTA of Ca^2+^ were selected. Interestingly, ionomycin (1 µM) treatment resulted in significant up-regulation of PKC-β expression in the infected macrophages compared to that of the uninfected one. However, treatment with a calcium ion chelator, EGTA (2 mM), abrogated the ionomycin induced enhancement of PKC-β expression in the infected macrophages. On the other hand, when both EGTA and ionomycin were used, no significant enhancement of the PKC-β expression in the infected macrophages (Fig. 3B) was observed. In contrast, atypical PKCs (PKC-ζ) exhibited a different type of protein expression. In this case, it was found that PKC-ζ expression significantly up-regulated in infected macrophages as well as in EGTA (2 mM) treated infected macrophages compared to that of the uninfected control macrophages. In contrast, treatment with ionomycin (1 µM) or ionomycin with EGTA resulted in a significant down regulation of PKC-ζ expression in the infected macrophages (Fig. 3B). Similar changes were also observed in the mRNA expression (Fig. 3A). All these findings clearly demonstrated that intracellular Ca^2+^ plays a significant role in regulating the protein as well as the mRNA expression of different PKC isotypes in *Leishmania donovani* infected macrophages.

Previous studies revealed that ionomycin treatment led to an enhancement of intracellular calcium release inside the cells, which can be modulated by the addition of EGTA [19]. Therefore, we intended to investigate the role of calcium ATPases in regulating the enhanced intracellular calcium following ionomycin treatment in *Leishmania donovani* infected macrophages. The infected and control macrophages were treated with 1 µM of ionomycin and EGTA (2 mM). When ionomycin (1 µM) was used, it was found that the mRNA expression of both the PMCA4 and SERCA3 significantly augmented in the infected macrophages compared to that of the untreated one. Interestingly, treatment with EGTA or EGTA+ionomycin resulted in a sharp decline in the PMCA4 and SERCA3 mRNA expression in the infected macrophages (Fig. 3B). The expression of calcium ATPases was found to be similar in the western blot (protein expression) (Fig. 3A) analysis also. Therefore, our findings suggested that PMCA4 and SERCA3 play an important role in the regulation of intracellular Ca^2+^ concentrations in macrophages during *Leishmania donovani* infection.

### Effect of ionomycin and EGTA on cytokine release pattern of Leishmania infected macrophages

**Figure 4:**
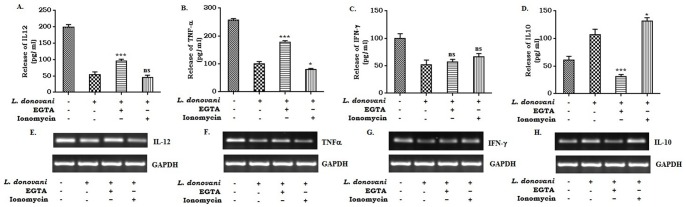
Effect of EGTA and ionomycin on the expression of pro- and anti-inflammatory cytokines in L. donovani infected macrophages. Macrophages (2×10^6^ cells) were infected with *L. donovani* (1∶10) and incubated at 37°C in the presence of 2 mM EGTA and/or 1 µM ionomycin. The cell supernatants were collected after 24 hr of infection. Cytokine assay was carried out by ELISA (A, B, C and D). Data represent means ± SD for 3 sets of experiments ***P<.001, *P<.05 and ns = non-significant for the comparison with infected one. In a parallel experiment, EGTA or Ionomycin treated infected cells were collected in Trizol for RNA extraction and semi-quantitative RT-PCR was performed. IL-12, TNF-α, IFN-γ, IL-10 and GAPDH PCR products were resolved on an agarose gel (1.5%) and quantified densitometrically using lab software as described in Methods. Data is from one representative experiment performed at least three times (E, F, G, and H).

Recent findings suggested the involvement of PKC-β in the induction of various pro-inflammatory cytokine expressions in macrophages [3]. It is well known that PKC-β activity is strictly dependent upon the intracellular calcium ion concentration [17]. Therefore, we investigated the involvement of calcium in the regulation of various pro- and anti-inflammatory cytokines in *Leishmania donovani* infected macrophages. Alteration in the expression level of cytokines as evident from ELISA and mRNA analyses in presence ionomycin/EGTA suggested the role of calcium (Fig. 4). A significant enhancement in IL-10 (Fig. 4D and H) expression along with concomitant decrease in the expression of IL-12 (Fig. 4A and E) and TNF-α (Fig. 4B and F) in infected macrophages was observed at 20 hr post ionomycin treatment. Interestingly, EGTA treatment completely abrogated the ionomycin induced expression of IL-10 in the infected macrophages (Fig. 4D and H). However, the IL-12 (Fig. 4A and E) and TNF-α (Fig. 4B and F) expression in infected macrophages were further augmented following EGTA treatment. On the contrary, ionomycin or EGTA treatment exhibited no significant change in the expression of IFN-γ (Fig. 4C and G) in the infected macrophages. Therefore, our result indicated that enhanced intracellular calcium positively regulated the expression of anti-inflammatory cytokine IL-10 along with significant down regulation of the pro-inflammatory cytokines- IL-12 and TNF-α expression in *Leishmania donovani* infected macrophages.

### Effect of Ca^+2^–ATPases on parasite burden

**Figure 5:**
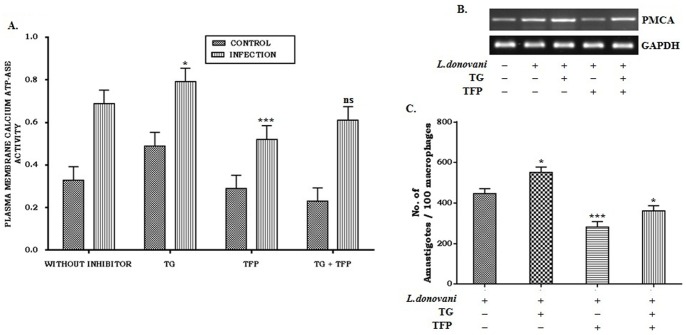
Effect of inhibitors on plasma membrane Ca^2+^-ATPases during Leishmania infection. Macrophages were treated with thapsigargin (TG, 1 µM) and trifluoperazine (TFP, 10 µM) followed by infection with *L.donovani* in 1∶10 ratio. Unbound parasites were washed off after 4 hr and further incubated for 20 hr. Cells were collected, plasma membrane was isolated and Ca^2+^-ATPase activity was measured. (A). In a similar experiment, inhibitor treated and infected macrophages were collected in Trizol for RNA extraction and semi-quantitative RT-PCR were performed (B). Macrophages were seeded on 8 well chamber slides (10,000 cells/well) and treated with inhibitors followed by infection with *L.donovani* in a 1∶10 ratio. Cells were incubated for 24 hr, fixed with chilled methanol, and stained with Giemsa. Amastigotes were then microscopically counted. ***P<.001, *P<.05 and ns = non-significant for the comparison with infected one (C). Data represented means ± SD from three different experiments (A and C). Result shown in B is a representative one from three different experiments.

Calcium appears to be vital in *Leishmania* apoptosis. It has been also established that more parasites die in the presence of Ca^2+^ than in its absence. Therefore, we focused on the parasitic load of the infected macrophages. In these experiments, macrophages were infected as described in Materials and Methods. Figure 5C shows that parasitic load was significantly increased in presence of thapsigargin; whereas a sharp decline was observed in presence trifluoperazine in comparison to the control. When both inhibitors were used parasitic load was found to be lower compared to the control.

From the foregoing study it is evident that macrophage contains both SERCA and PMCA. We, therefore, examined the level of PMCA activity when SERCA was inhibited by its specific inhibitor namely thapsigargin (Tg). The result presented here revealed that the PMCA activity was indeed increased under thapsigargin induced SERCA inhibition (Fig. 5A). This was further evident from the mRNA expression of PMCA in presence of this inhibitor (Fig. 5B).

## Discussion

Cytoplasmic Ca^2+^ acts as a second messenger controlling many different aspects of cellular function [20]. Intracellular organelles, like ER and mitochondria, play critical roles in maintaining Ca^2+^ homeostasis by regulating certain Ca^2+^ channels and pumps [20], like sarco-endoplasmic reticulum Ca^2+^-ATPases (SERCA). Since intracellular organelles are limited by their storage capacity, long-term calcium homeostasis is regulated solely by the plasma membrane Ca^2+^-ATPases (PMCA) [21].

During leishmaniasis, intracellular Ca^2+^ concentration is found to be enhanced [3], [22]. Despite this, Ca^2+^-dependent PKC activation is impaired during leishmaniasis [4], [8], [10]. Following this information, we intended to study the involvement of Ca^2+^ and Ca^2+^-ATPases during *Leishmania donovani* infection. Consistent with the previous reports, we observed an upregulation in the intracellular Ca^2+^ concentration in *Leishmania* infected macrophages, which was further increased by trifluoperazine (TFP), a PMCA4 inhibitor. Interestingly, SERCA3 inhibition by thapsigargin (Tg) resulted in a transient decline in intracellular Ca^2+^ level. It was also observed that both ATPases (SERCA3 and PMCA4) expressions were augmented during the course of infection. These expressions were further enhanced by ionomycin treatment. However, EGTA abrogated their expression, implying that excess Ca^2+^ from the infected cells might be pumped out via the activation of Ca^2+^-ATPases.

Intracellular Ca^2+^ concentration significantly affect the activation of classical PKCs. During visceral leishmaniasis, Ca^2+^-dependent PKC activation is impaired, whereas PKC-ζ, (atypical PKC), is induced [4], [8], [10]. In our study, we observed that in *Leishmania* infected macrophages, the impaired PKC-β (classical PKC) expression was induced by ionomycin treatment, whereas PKC-ζ expression was decreased. This may suggest that the differential function of cellular Ca^2+^ may be involved in the impairment of Ca^2+^ -dependent PKCs during pathogenesis [23]–[25].

It has been reported that Ca^2+^ signaling effectively modulate the pro- and anti-inflammatory cytokine production during pathogenesis [26]–[29]. Thus, moderation of cellular Ca^2+^ signaling in macrophages seems to be one of the major strategies employed by the *Leishmania* parasite for its survival within the macrophages. In the present study, ionomycin treatment did not change the enhanced release of IL-10 and decreased release of IL-12 and TNF-α in *L. donovani* infected macrophages, may be that ionomycin play different role in the regulation of pro- and anti-inflammatory cytokines [26]–[29]. No significant change was observed in case of IFN-γ expression. From these findings it can be assumed that the Ca^2+^ generated during *Leishmania* infection, induces Th2 type immune response in favour of parasitemia.

From the foregoing study it has been demonstrated that PMCA4 activity is significantly elevated in thapsigargin treated cells along with the enhancement of parasite burden, while the inhibition of PMCA4 activity showed significant reduction under this condition. It is reported that Ca^2+^ signaling is involved in the transformation of promastigotes to axenic amastigotes of *Leishmania donovani* [30], whereas increased intracellular Ca^2+^ is responsible for apoptosis of intracellular *Leishmania donovani* amastigotes [31]–[33]. Thus, our findings suggested that plasma membrane Ca^2+^-ATPases helps in pumping out the extra Ca^2+^ from the intracellular milieu of *Leishmania* infected cells, which in-turn helps the survivility of *Leishmania* parasites within the host cells.

## References

[1] Murray HW, Spitalny GL, Nathan CF (1985) Activation of mouse peritoneal macrophages in vitro and in vivo by interferon-gamma. J Immunol 134: 1619–1622. 3918107

[2] Wilmann M, Gautel M, Mayans O (2000) Activation of calcium/calmodulin regulated kinases. Cell Mol Biol 46(5): 883–94. 10976872

[3] Olivier M, Gregory DJ, Forget G (2005) Subversion mechanisms by which *Leishmania* parasites can escape the host immune response: A signaling point of view. Clin Microbiol Rev 18(2): 293–305. 15831826 10.1128/CMR.18.2.293-305.2005 PMC1082797

[4] Descoteaux A, Matlashewski G (1989) c-fos and tumor necrosis factor gene expression in *Leishmania donovani*-infected macrophages. Mol Cell Biol 9: 5223–7. 2513483 10.1128/mcb.9.11.5223-5227.1989 PMC363676

[5] Nishizuka (1988) Studies and prospectives of protein kinase C in signal transduction. Nippon Ketsueki Gakkai Zasshi 51(8): 1321–6. 3247812

[6] Parker PJ, Kour G, Marais RM, Mitchell F, Pears C, et al (1989) Protein kinase C-a family affair. Mol Cell Endocrinol 65(1–2): 1–11. 2673888 10.1016/0303-7207(89)90159-7

[7] Bhattacharyya S, Ghosh S, Sen P, Roy S, Majumdar S (2001) Selective impairment of protein kinase C isotypes in murine macrophage by *Leishmania donovani* . Mol Cell Biochem 216(1–2): 47–57. 11216863 10.1023/a:1011048210691

[8] Ghosh S, Bhattacharyya S, Das S, Raha S, Maulik N, et al (2001) Generation of ceramide in murine macrophages infected with *Leishmania donovani* alters macrophage signaling events and aids intracellular parasitic survival. Mol Cell Biochem 223(1–2): 47–60. 11681721 10.1023/a:1017996609928

[9] Yano S, Tokumitsu H, Soderling TR (1998) Calcium promotes cell survival through CaM-K kinase activation of the protein-kinase-B pathway. Nature 396: 584–587. 9859994 10.1038/25147

[10] Schmitt JM, Wayman GA, Nozaki N, Soderling TR (2004) Calcium activation of ERK mediated by calmodulin kinase I. J Biol Chem. 279(23): 24064–72. 10.1074/jbc.M401501200 15150258

[11] Pandey D, Gratton JP, Rafikov R, Black SM, Fulton DJR (2011) Calcium/Calmodulin-dependent kinase II mediates the phosphorylation and activation of NADPH oxidase 5. Molecular Pharmacology 80(3): 407–415. 21642394 10.1124/mol.110.070193 PMC3164331

[12] Sarkadi B, Price EM, Boucher RC, Germann UA, Scarborough GA (1992) Expression of the human multidrug resistance cDNA in insect cells generates a high activity drug-stimulated membrane ATPase. J Biol Chem 267(7): 4854–8. 1347044

[13] Sambrook J, Fritsch EF, Maniatis T (1989) Molecular cloning: A laboratory manual. Cold spring harbor, NY: Cold Spring Harbor Laboratory Press.

[14] Robertson MJ, Cameron C, Lazo S, Cochran KJ, Voss SD, et al (1996–1997) Costimulation of human natural killer cell proliferation: role of accessory cytokines and cell contact-dependent signals. Nat Immun 15(5): 213–26. 9390270

[15] Mishina YV, Krishna S, Haynes RK, Meade JC (2007) Artemisinins inhibit *Trypanosoma cruzi* and *Trypanosoma brucei* rhodesiense in vitro growth. Antimicrob Agents Chemother 51(5): 1852–4. 17339374 10.1128/AAC.01544-06 PMC1855540

[16] Bhatnagar K, Singh VP (2004) Ca^2+^ dependence and inhibitory effects of trifluoperazine on plasma membrane ATPase of *Thermoactinomyces vulgaris* . Curr Microbiol 49(1): 28–31. 15297926 10.1007/s00284-003-4261-8

[17] Christianse NO, Larsen CS, Esmann V (1988) A study on the role of protein kinase C and intracellularcalcium in the activation of superoxide generation. Biochimica et Biophysica Acta 971: (317–324). 10.1016/0167-4889(88)90147-4 2844293

[18] Violin JD, Zhang J, Tsien RY, Newton AC (2003) A genetically encoded fluorescent reporter reveals oscillatory phosphorylation by protein kinase C. J Cell Biol. 161(5): 899–909. 10.1083/jcb.200302125 PMC2172956 12782683

[19] Kress M, Distler C (2004) Differences in calcium signalling in rat peripheral sensory neurons. Neuroscience Letters 354: 127–130. 14698455 10.1016/j.neulet.2003.10.003

[20] Lytton J, Westlin M, Burk SE, Shull GE, MacLennan DH (1992) Functional comparisons between isoforms of the sarcoplasmic or endoplasmic reticulum family of calcium pumps. Journal of Biological Chemistry 267: 14483–14489. 1385815

[21] Benaim B, Garcia CR (2011) Targeting calcium homeostasis as the therapy of Chagas' disease and leishmaniasis - a review. Trop Biomed 28(3): 471–81. 22433874

[22] Lu HG, Zhong L, Chang KP, Docampo R (1997) Intracellular Ca^2+^ pool content and signaling and expression of a calcium pump are linked to virulence in *Leishmania mexicana* amazonesis amastigotes. The Journal of Biological Chemistry 272: 9464–9473. 9083086 10.1074/jbc.272.14.9464

[23] Buchmuller-Rouiller Y, Mauel J (1991) Macrophage activation for intracellular killing as induced by calcium ionophore. Correlation with biologic and biochemical events. J Immunol 146(1): 217–23. 1898600

[24] Awasthi A, Mathur RK, Saha B (2004) Immune response to *Leishmania* infection. Ind J Med Res 119: 238–258. 15243162

[25] Goto Y, Sanjoba C, Asada M, Saeki K, Onodera T, et al (2008) Adhesion of MRP8/14 to amastigotes in skin lesions of *Leishmania major* -infected mice. Experimental Parasitology 119(1): 80–6. 18272153 10.1016/j.exppara.2007.12.019

[26] Lehmann MH, Berg H (1998) Interleukin-10 expression is induced by increase of intracellular calcium levels in the monocytic cell line U937. Eur J Physiol 435: 868–870. 10.1007/s004240050596 9518518

[27] Faries MB, Bedrosian I, Xu S, Koski G, Roros JG, et al (2001) Calcium signaling inhibits interleukin-12 production and activates CD831 dendritic cells that induce Th2 cell development. Blood 98: 2489–2497. 11588047 10.1182/blood.v98.8.2489

[28] Bhattacharyya S, Ghosh S, Jhonson PL, Bhattacharya SK Majumdar S (2001) Immunomodulatory role of interleukin-10 in visceral leishmaniasis: defective activation of protein kinase c-mediated signal transduction events. Infect Immun 69(3): 1499–1507. 11179319 10.1128/IAI.69.3.1499-1507.2001 PMC98048

[29] Peiretti F, Alessi MC, Henry M, Anfosso F, Vague IJ, et al (1997) Intracellular calcium mobilization suppresses the TNF-α–stimulated synthesis of PAI-1 in human endothelial cells indications that calcium acts at a translational level. Arteriosclerosis Thrombosis and Vascular Biology 17: 1550–1560. 10.1161/01.atv.17.8.1550 9301635

[30] Prasad A, Kaur S, Malla N, Ganguly NK, Mahajan RC (2001) Ca^2+^ signaling in the transformation of promastigotes to axenic amastigotes of *Leishmania donovani* . Molecular and Cellular Biochemistry 224: 39–44. 11693198 10.1023/a:1011965109446

[31] Sudhandiran G, Shaha C (2003) Antimonial-induced increase in intracellular Ca^2+^ through non-selective cation channels in the host and the parasite is responsible for apoptosis of intracellular *Leishmania donovani* amastigotes. J Biol Chem 278(27): 25120–32. 12707265 10.1074/jbc.M301975200

[32] Lanza H, Cardoso SRA, Silva AG, Napolitano DR, Espindola FS, et al (2004) Comparative effect of ion calcium and magnesium in the activation and infection of the murine macrophage by *Leishmania major.* . Biol Res 37(3): 385–93. 15515964 10.4067/s0716-97602004000300004

[33] Shaha C (2006) Apoptosis in *Leishmania* species and its relevance to disease pathogenesis. Indian J Med Res 123: 233–244. 16778307

