# Expression of Concern: Regulation of PKC Mediated Signaling by Calcium during Visceral Leishmaniasis

**DOI:** 10.1371/journal.pone.0350440

**Published:** 2026-06-02

**Authors:** The PLOS One Editors

After this article [1] was published, concerns were raised regarding the results presented in Fig 3 and the statistical analyses. Specifically:

- The Fig 3B GAPDH panel in [1] appears similar to Fig 1B GAPDH lanes 1–5 in [2,3] despite being used to represent different experimental conditions.
- The Statistical analysis subsection of the Materials and Methods section only reports that data were analyzed using Student’s *t* test; however, multiple experiments in this article [1] appear to present more than two conditions, suggesting a multivariate methodology should have been used.
- Contrary to the article’s Data Availability statement, the original raw data files supporting the article’s results were not provided with the article.

The first author disagreed with the journal’s observations that the results in the Fig 3B panel in [1] appear similar to results in Fig 1B in [2,3] and stated that the original image data for this figure are no longer available. In the absence of these data, this concern cannot be resolved and the reliability of the associated quantified results cannot be verified.

Regarding the statistical analyses, the first author stated that the Materials and Methods section is incomplete, and that for Figs 4 and 5, one-way ANOVA was performed. They state that the original underlying quantitative data for these figures are no longer available. In the absence of these data, PLOS cannot confirm the methodology used for the analysis and the reliability of the results reported in Figs 4 and 5.

The first author stated the original underlying data cannot be provided as the laboratory has closed since the publication of the article [1], and the original files are no longer accessible. As the original raw data files supporting the article’s results have not been made available, this article [1] does not comply with the PLOS Data Availability policy in place at the time this article was submitted**.**

The *PLOS One* Editors issue this Expression of Concern to inform readers of the above unresolved concerns and that the results presented in Figs 3B, 4, and 5 should be interpreted with caution.
